# Periodontal treatment and subsequent clinical outcomes and medical care costs: A retrospective cohort study

**DOI:** 10.1371/journal.pone.0290028

**Published:** 2023-08-14

**Authors:** Bryan S. Michalowicz, Jeffrey P. Anderson, Thomas E. Kottke, Steven P. Dehmer, Donald C. Worley, Sheryl Kane, Sarah Basile, D. Brad Rindal

**Affiliations:** 1 HealthPartners Institute, Bloomington, Minnesota, United States of America; 2 Genesis Research, Hoboken, New Jersey, United States of America; National Dental Research Institute Singapore / Duke NUS Medical School Singapore, SINGAPORE

## Abstract

**Introduction:**

Periodontitis is a common oral disease associated with coronary artery disease (CAD), cerebrovascular disease (CBVD) and type 2 diabetes (T2D). We studied if periodontitis treatment improves clinical outcomes and reduces medical care costs in patients with CAD, CBVD or T2D.

**Methods:**

We used clinic records and claims data from a health care system to identify patients with periodontitis and CAD, CBVD or T2D, and to assess periodontal treatments, hospitalizations, medical costs (total, inpatient, outpatient, pharmacy), glycated hemoglobin, cardiovascular events, and death following concurrent disease diagnoses. We compared clinical outcomes according to receipt of periodontal treatment and/or maintenance care in the follow-up period, and care costs according to treatment status within one year following concurrent disease diagnoses, while adjusting for covariates. The data were analyzed in 2019–21.

**Results:**

We identified 9,503 individuals, 4,057 of whom were in the CAD cohort; 3,247 in the CBVD cohort; and 4,879 in the T2D cohort. Patients who were selected and elected to receive treatment and maintenance care were less likely to be hospitalized than untreated individuals (CAD: OR = 0.71 (95% CI: 0.55, 0.92); CBVD: OR = 0.73 (0.56, 0.94); T2D: OR = 0.80 (0.64, 0.99)). Selection to treatment and/or maintenance care was not significantly associated with cardiovascular events, mortality, or glycated hemoglobin change. Total care costs did not differ significantly between treated and untreated groups over 4 years. Treated patients experienced lower inpatient costs but higher pharmacy costs.

**Conclusions:**

Patients with periodontitis and CAD, CBVD or T2D who were selected and elected to undergo periodontal treatment or maintenance care had lower rates of hospitalizations, but did not differ significantly from untreated individuals in terms of clinical outcomes or total medical care costs.

## Introduction

Periodontitis is a chronic multifactorial inflammatory disease associated with dysbiotic plaque biofilms and characterized by progressive destruction of the tooth-supporting apparatus [1]. It is highly prevalent and a major cause of tooth loss in adults [2, 3]. Periodontitis has been associated with myriad non-oral diseases, including coronary artery disease (CAD), cerebrovascular disease (CBVD), and type 2 diabetes (T2D) [4]. The putative mechanisms linking these conditions include immune responses to bacteria that have translocated through inflamed periodontal tissues to distant organs, and the systemic action of locally (orally)-produced inflammatory mediators [5]. Thus, treatment of periodontitis has been studied as a means of mitigating the incidence and progression of non-oral diseases.

Periodontal treatment in adults with T2D yields modest, short-term improvements in glycated hemoglobin (HbA1c), although the sustainability of this effect is uncertain [6]. Treatment may improve brachial artery compliance and endothelial cell activity but has not been shown to improve clinical outcomes in patients with cardiovascular disease [7–9]. To date, large randomized controlled trials are lacking for CAD and CBVD. Periodontitis, but not its treatment, is consistently associated with increases in all-cause mortality and death due to CAD and CBVD [10].

Health insurance claims data have been used to explore relationships between periodontitis, its treatment and non-oral diseases [11–15]. This approach, however, is limited by concerns with misclassification bias and confounding [16]. Use of integrated medical and dental records can circumvent some of the limitations inherent in claims data [17].

Few studies have examined the relationship between periodontal treatment and medical care costs [18–22]. Jeffcoat et al. reported substantial care costs savings for CAD, CBVD and T2D patients following periodontal treatment [20]. Although some have advocated for Medicare to include dental services, in part to help reduce medical care costs for seniors, at present there is insufficient evidence that periodontal treatment reduces medical care costs [23–25].

We sought to address some of the shortcomings of prior investigations by leveraging multiple data systems. We used medical and dental clinic records and insurance plan and claims data and a retrospective cohort study design to explore associations between receipt of periodontal treatment and clinical and care cost outcomes, while controlling for medical and dental variables generally not available in claims databases alone.

## Materials and methods

### Study sample

We used clinical records and insurance claims data from patients seen in HealthPartners clinics. HealthPartners provides care and insures more than 1 million members through its hospitals and medical and dental clinics in Minnesota and Wisconsin. We searched records from January 2004 through June 2017, inclusive, to identify patients who: 1) were > = 18 years of age; 2) had ≥1 periodontal disease diagnosis code and ≥1 diagnosis code(s) for coronary artery disease (CAD; ICD-9 codes 410–414; ICD-10 codes I20-I25), cerebrovascular disease (CBVD; ICD-9 codes: 433–436, 437.1, 437.4, 437.6, 437.9, 438; ICD-10 codes: I60-I69), or type 2 diabetes (T2D; ICD-9 codes: 250, 357.2, 362.0, 366.41; ICD-10 codes: E11); 3) had not opted out of research; and 4) had continuous (allowing gaps ≤30 days) medical, pharmacy, and dental insurance coverage for at least one year prior to, and one year beyond the date at which both a qualifying medical and periodontitis diagnoses were first observed. HealthPartners’ periodontal diagnosis codes map to the 1999 American Academy of Periodontology’s classifications [26]. We classified an individual based on their most severe periodontal disease diagnosis (i.e., severe over moderate over slight). Individuals were also categorized based on the extent of their most severe disease: generalized, affecting roughly ≥30% of the remaining teeth; or localized, affecting less than 30% of remaining teeth. Finally, the extent of gingival inflammation (i.e., the gingiva was visibly inflamed or bled when probed) was similarly scored as generalized or localized based on the above criteria.

### Exposure and covariates

Periodontal treatment was ascertained based on American Dental Association’s Current Dental Treatment (CDT) codes, abstracted from dental records: D4341, D4342, D4355, D4210, D4211, D4240, D4241, D4245, D4260, D4261, D4263-7, D4264, D4274 and D4910. D4341, D4342 and D4355 denote non-surgical procedures (scaling or “deep cleaning”). D4910 denotes a maintenance procedure (cleaning) that typically follows treatment. The other codes denote surgical procedures. We categorized individuals as having received active therapy alone (non-surgical and/or surgical procedures), active therapy plus maintenance care, maintenance care only, or no care following the index date. Extractions were not considered as periodontal treatment, although tooth count (from dental records) was considered as a baseline covariate and updated with subsequent extractions.

We abstracted demographic information, laboratory results, vital signs, and smoking history from medical records. Smoking status [current (some days, every day, heavy smoker, light smoker); former (in the past but not currently); never (never)] was determined from self-reports at baseline (I.e., the index date). We computed the Charlson Co-Morbidity Index from ICD-9 or -10 codes present at the index date/baseline [27]. Dental insurance coverage was ascertained from health plan data.

### Outcomes

We tracked clinical outcomes and medical care costs after an individual’s index date, which was the earliest date the individual had both a periodontitis and CAD, CBVD or T2D diagnosis. For the clinical outcomes, individuals were followed (retrospectively) from their index date until death, censoring due to discontinued enrollment in insurance plans, or the end of the follow-up period. Medical care costs were tracked starting at the index date and followed for up to 4 years following the index date. Hospitalizations, total medical care costs, and change in HbA1c (for the T2D cohort) were the pre-specified primary outcomes. Secondary outcomes included cardiovascular-related hospitalizations, composite cardiovascular events, all-cause death, and component medical care costs (i.e., inpatient, outpatient, and pharmacy costs). Inpatient hospitalizations were determined from insurance claims, cardiovascular-related hospitalizations from diagnosis-related group (DRG) codes (215–320), all-cause death from medical records and Minnesota Death Records, composite cardiovascular events (myocardial infarction, stroke, angioplasty and bypass graft procedure) from ICD, CPT or HCPCS codes (Table 1), and HbA1c from medical records, for patients with T2D.

**Table 1:** Components of the composite cardiovascular study outcome.

DESCRIPTION	SOURCE	CODE
Death (Any Cause)	EHR, MN Death Index	
Myocardial Infarction	ICD-9 Diagnoses	410.xx
	ICD-10 Diagnoses	I21
Stroke	ICD-9 Diagnoses	362.3x, 430, 431, 433.x1, 434.x1, 435.x, 436
	ICD-10 Diagnoses	G45.0, G45.1, G45.2, G45.3, G45.8, G45.9, H34.1x, I60.1x, I60.2, I60.3x, I60.8, I60.9, I61.0, I61.1, I61.2, I61.4, I61.5, I61.6, I61.8, I61.9, I63.0xx, I63.1xx, I63.2xx, I63.3xx, I63.4xx, I63.5xx, I63.8x, I63.9
Angioplasty Procedure	CPT Procedures	92920, 92921, 92924, 92929, 92933, 92937, 92941, 92943
	HCPCS Procedures	C9600, C9601, C9602, C9604, C9606, C9607
	ICD-9 Procedures	00.66
Bypass Procedure	CPT Procedures	33510, 33511, 33512, 33513, 33514, 33516, 33517, 33518, 33519, 33521, 33522, 33523, 33533, 33534, 33535, 33536

We assessed utilization of non-dental healthcare services from insurance claims. Specifically, we classified outpatient care using Current Procedural Terminology, 4th Edition, and Healthcare Common Procedure Coding System procedure codes; inpatient care using Medicare Severity DRG coding; and pharmacy utilization for medication fills using 11-digit National Drug Codes. Overall costs consisted of outpatient (utilization related to primary care, urgent care, emergency care, specialty care, laboratory procedures, and radiology), inpatient, and pharmacy costs.

We converted medical and pharmacy claims to dollar amounts using Total Care Relative Resource Values^™^ (TCRRVs) [28]. TCRRVs are a nationally standardized set of pricing measures that have been endorsed by the National Quality Forum and are derived from Centers for Medicare and Medicaid Services (CMS) relative value units (RVUs) [29]. We used 2017 TCRRVs to convert all claims costs to 2017 U.S. dollars.

### Statistical analyses

For analyses of clinical outcomes, we constructed a longitudinal dataset with patient-month as the unit of analysis. Outcome, treatment, and covariate values were carried forward and updated as new measures were available. Cohort-specific data sets were drawn from this master set. Patients were followed (retrospectively) from baseline until death, censoring due to discontinued enrollment in insurance plans, or the end of the follow-up period.

We tabulated descriptive statistics and evaluated bivariate associations using Kruskal-Wallis or chi-square tests as appropriate. We constructed generalized linear models (GLM), with an independent covariance structure for repeated measures, assuming a binomial distribution with a logit link function (except for HbA1c, a continuous outcome for which a normal distribution and identity link were specified). The GLM methods we used were equivalent to Cox survival models using pooled logistic regression [30–32].

Additionally, we estimated time-dependent, stabilized inverse probability weights (IPW) for likelihood of active periodontal treatment and censoring using the R package ‘ipw’. For the IPW weighting, a binomial family with a logit link was used. Time-related variables included: Age and Years Since 1st Diagnosis of Periodontal Disease. Time-varying variables included: Charlson Comorbidity Index, Tooth Count, Cumulative Months of Continuous Enrollment in Dental Plan, Generalized Periodontal Disease, Severe Periodontal Disease, Comprehensive Dental Insurance, Current Smoker, and History of Coronary Artery Disease. Time-invariant variables included: Male, Non-White.

We included covariates for the treatment and censoring models if they were specified a priori or were selected via LASSO (least absolute shrinkage and selection operator) [33, 34]. Table 2 contains additional details on these propensity models. In these models we considered an individual’s periodontitis diagnosis (slight, moderate or severe), extent of disease (generalized or localized) and extent of gingival inflammation (generalized or localized) as separate covariates. Final adjusted models were weighted by the time-dependent product of treatment and censoring weights. We truncated weights at the 1^st^ and 99^th^ percentiles to attenuate potential influence of extreme values, and report adjusted effect estimates as odds ratios (beta estimates for A1C) with 95% confidence intervals.

**Table 2:** Propensity of active periodontal therapy model.

VARIABLE	β	P-VALUE
Intercept	-11.503	<0.001
Age, in Years (continuous)^a^^,^^b^	0.081	<0.001
Age squared	-0.001	<0.001
Charlson Comorbidity Index (continuous)^a^^,^^c^	-0.014	0.399
Tooth Count (continuous)^a^^,^^d^	0.204	<0.001
Tooth Count squared	-0.004	<0.001
Years Since 1st Diagnosis of Periodontal Disease (PD)^b^ (continuous)	-0.422	<0.001
PD Years squared	0.026	<0.001
Cumulative Months of Continuous Enrollment in Dental Plan^b^	-0.005	<0.001
Male (y/n)^a^	0.068	0.399
Non-White (y/n)^a^	0.288	0.002
Generalized Periodontal Disease^a^^,^^e^	0.918	<0.001
Severe Periodontal Disease^a^^,^^f^	0.954	<0.001
Generalized gingival inflammation^a^^,^^g^	1.330	<0.001
Comprehensive Dental Insurance^a^^,^^h^	0.695	<0.001
Current Smoker (y/n)^a^^,^^i^	-0.171	0.151
History of Coronary Artery Disease (y/n)	-0.234	0.005

^a^Specified a priori

^b^Per 1-year increase

^c^Per 2-unit increase

^d^Per 1-tooth increase

^e^Denotes disease affecting > 30% of remaining teeth, and versus localized disease

^f^Versus slight or moderate periodontitis

^g^Denotes widespread visible inflammation or gingival bleeding (~≥30% of tooth sites), as judged by the clinician, and versus localized gingival inflammation

^h^Versus plans covering preventive services only (i.e., exclude a periodontal therapy benefit)

^i^Versus never, former, or missing, and based on self-reports at baseline

Next, we calculated cumulative total and component costs at 1, 2, 3, and 4 years from baseline, which we modeled as a function of baseline covariates. We truncated extreme values at the 99^th^ percentile. For cost models, we considered individuals as treated if they had codes for active periodontal therapy (active treatment with or without maintenance care) by the end of year 1. Values for total, outpatient, and pharmacy cost outcomes were log-transformed and modeled assuming a normal distribution on the log scale. Because of a high proportion of zero values (76.7% of study patients did not incur inpatient costs in the 1^st^ year, and 28.1% in 4 years), and a right-skewed distribution, we modeled inpatient costs using a 2-stage process [35]. We report estimates as the difference in mean costs by treatment status, with 95% confidence intervals.

We examined the timing of an individual’s initial periodontal and medical diagnoses and considered patients as “incident” cases if the first periodontitis diagnosis appeared after the medical diagnosis, and “prevalent” if the periodontitis diagnosis preceded the initial medical diagnosis of interest. We conducted stratified analyses separately within each disease cohort (“prevalent” and “incident” cases). These results were qualitatively similar to those using the entire cohort and are not reported.

We compiled and analyzed the data in 2019–21 and used SAS v9.4 (SAS Institute, Cary, NC) and R v4.0.3 (cran.r-project.org). We report two-sided p-values.

### Ethics statement

This research was approved by the HealthPartners IRB. Individuals who opted out of using their medical record data for research were not included in this study. Only medical record data was utilized so consenting of individuals was not required. Prior to the release of the data sets to investigators, private health information was removed and a random identification number was assigned.

## Results

Our initial search identified 23,935 adult patients with a diagnosis of periodontitis and CAD, CBVD or T2D. Of these, 133 had opted out of research and 14,299 did not have continuous insurance coverage, yielding a final sample of 9,503 (4,057 with CAD; 3,247 with CBVD; and 4,879 with T2D). About one-fourth (26.3%) of individuals were included in two cohorts and 8.0% in all three. Table 3 summarizes baseline characteristics by cohort and treatment status. About three-quarters of individuals in each cohort were White. In all cohorts, those receiving active periodontal therapy (non-surgical or surgical treatment) were more likely than others to be male, non-White, and current smokers. Treated individuals had fewer comorbidities and were more likely to have active, generalized, and advanced periodontal disease, and comprehensive dental insurance (p<0.001 for all cohorts).

**Table 3:** Baseline characteristics of patients with periodontal disease by cohort and active treatment status.

	CAD^a^			CBVD^b^			T2D^c^		
	Active Treatment			Active Treatment			Active Treatment		
	No	Yes	P-Value	No	Yes	P-Value	No	Yes	P-Value
Total (N)	3,517	540		2,817	430		3,942	937	
Age (mean ± SD, in years)	71.6 ± 11.9	65.3 ± 11.8	**<0.001** ^d^	73.6 ± 12.7	66.1 ± 12.5	**<0.001**	64.9 ± 13.9	57.1 ± 13.2	**<0.001**
Male (%)	55.3	63.7	**<0.001**	45.3	49.8	0.087	50.6	57.6	**<0.001**
White (%)	88.7	70.0	**<0.001**	88.4	65.4	**<0.001**	78.3	50.0	**<0.001**
Black (%)	3.8	15.2		3.8	17.4		8.1	26.8	
Asian (%)	2.0	5.7		2.6	7.4		5.0	11.2	
Other/Unknown (%)	5.5	9.1		5.3	9.8		8.7	12.1	
CAD (%)	100.0	100.0	-	48.7	45.4	0.196	24.0	17.0	**<0.001**
CBVD (%)	23.0	20.0	0.136	100.0	100.0	-	13.9	8.1	**<0.001**
T2D (%)	31.2	34.3	0.150	28.0	32.3	0.076	100.0	100.0	-
Charlson Comorbidity Index (mean ± SD)	6.5 ± 3.4	5.6 ± 3.4	**<0.001**	7.2 ± 3.3	6.6 ± 3.4	**<0.001**	5.5 ± 3.3	4.2 ± 3.0	**<0.001**
Incident Periodontal Disease (%)	56.6	44.3	**<0.001**	42.7	31.6	**<0.001**	60.8	48.7	**<0.001**
Generalized Gingival Inflammation (%)	65.2	82.8	**<0.001**	64.9	82.8	**<0.001**	71.6	87.4	**<0.001**
Generalized Periodontal Disease (%)	16.0	38.7	**<0.001**	16.4	38.8	**<0.001**	16.3	39.7	**<0.001**
Severe Periodontal Disease (%)	18.0	44.3	**<0.001**	19.0	39.3	**<0.001**	18.9	38.2	**<0.001**
Tooth Count (mean ± SD)	23.8 ± 6.5	25.0 ± 5.8	**<0.001**	23.8 ± 6.5	24.6 ± 6.0	0.029	24.4 ± 6.4	25.8 ± 6.4	**<0.001**
Current Smoker (%)	8.3	15.6	**<0.001**	8.7	17.0	**<0.001**	10.2	16.4	**<0.001**
Former Smoker (%)	46.5	44.1		44.5	40.9		38.0	32.8	
Never Smoker (%)	41.3	34.3		43.9	37.9		44.1	42.3	
Unknown Smoking Status (%)	3.9	6.1		2.9	4.2		7.8	8.5	
Comprehensive Dental Coverage (%)	63.4	85.4	**<0.001**	63.0	84.4	**<0.001**	71.5	92.2	**<0.001**

^a^Coronary artery disease

^b^Cerebrovascular disease

^c^Type 2 diabetes

^d^Bolded P values are significant at P<0.001

Mean follow-up for the clinical outcomes was 7.5 years in the CAD, 4.8 years in the CBVD, and 5.3 years in the T2D cohorts (maximum 13.9 years in all). For prevalent periodontal cases, the mean time to initial medical diagnosis was 6.4 years for CAD, 5.0 years for CVD, and 6.2 years for T2D. Excluding those who received maintenance care only, 16.1% of individuals in the CAD and CBVD cohorts, and 23.4% in the T2D cohort received non-surgical or surgical (i.e., active) periodontal therapy prior to the end of follow-up. Variables specified a priori that were significantly predictive of active treatment included age; race; commercial dental coverage; extent, severity, and active status of periodontal disease; and tooth count (Table 2). LASSO identified additional predictors, including history of CAD and cumulative time enrolled in a dental plan.

Table 4 lists unadjusted and IPW (adjusted) effect estimates for the clinical outcomes. In all cohorts and relative to no treatment, the combination of active periodontal treatment and maintenance care was associated with a lower risk of hospitalization for any cause, but not with cardiovascular-related hospitalization. There were 4.0, 5.0 and 2.6 deaths per 100 person-years of follow-up in the CAD, CBVD and T2D cohorts, respectively. Active treatment, alone or in combination with maintenance care, was inversely associated with mortality in all cohorts in unadjusted models. Following adjustments, effect estimates were strongly attenuated toward the null. Similarly, active treatment alone or in combination with maintenance care was significantly associated with the composite cardiovascular outcome in unadjusted but not adjusted analyses.

**Table 4:** Effect estimates for receipt of periodontal therapy and clinical outcomes.

	CAD^a^					CBVD^b^					T2D^c^				
	Events^d^	Unadjusted		Adjusted^e^		Events^d^	Unadjusted		Adjusted		Events^d^	Unadjusted		Adjusted	
		OR	95% CI	OR	95% CI		OR	95% CI	OR	95% CI		OR	95% CI	OR	95% CI
**Hospitalization**															
No Treatment	3,910 (2.2)	Referent		Referent		2,953 (2.3)	Referent		Referent		3,471 (1.7)	Referent		Referent	
Active Only	208 (1.6)	0.74	0.61, 0.90	0.72	0.56, 0.94	152 (1.6)	0.70	0.54, 0.90	0.83	0.63, 1.09	307 (1.2)	0.71	0.57, 0.88	1.08	0.80, 1.46
Maintenance Only	874 (2.1)	0.95	0.85, 1.07	0.93	0.82, 1.05	690 (2.3)	1.01	0.88, 1.16	1.05	0.91, 1.21	832 (1.9)	1.11	0.97, 1.26	1.07	0.92, 1.23
Active + Maintenance	314 (1.3)	0.60	0.50, 0.72	0.71	0.55, 0.92	232 (1.6)	0.68	0.57, 0.82	0.73	0.56, 0.94	446 (1.1)	0.66	0.56, 0.78	0.80	0.64, 0.99
**Cardiovascular Hospitalization**															
No Treatment	1,091 (0.6)	Referent		Referent		660 (0.5)	Referent		Referent		739 (0.4)	Referent		Referent	
Active Only	72 (0.6)	0.92	0.68, 1.25	0.85	0.60, 1.19	37 (0.4)	0.76	0.48, 1.20	0.93	0.57, 1.51	59 (0.2)	0.64	0.46, 0.90	0.83	0.58, 1.18
Maintenance Only	224 (0.5)	0.88	0.73, 1.05	0.84	0.69, 1.02	161 (0.5)	1.05	0.84, 1.32	1.15	0.92, 1.44	184 (0.4)	1.15	0.92, 1.43	1.11	0.89, 1.38
Active + Maintenance	97 (0.4)	0.67	0.52, 0.86	0.78	0.55, 1.12	45 (0.3)	0.60	0.42, 0.85	0.66	0.41, 1.07	95 (0.2)	0.66	0.48, 0.93	0.73	0.48, 1.09
**Mortality**															
No Treatment	688 (0.4)	Referent		Referent		621 (0.5)	Referent		Referent		517 (0.3)	Referent		Referent	
Active Only	16 (0.1)	0.32	0.20, 0.53	0.81	0.48, 1.36	19 (0.2)	0.41	0.26, 0.65	1.04	0.62, 1.75	24 (0.1)	0.37	0.25, 0.56	1.71	1.01, 2.92
Maintenance Only	122 (0.3)	0.76	0.63, 0.91	0.83	0.68, 1.02	113 (0.4)	0.79	0.65, 0.96	0.91	0.74, 1.13	92 (0.2)	0.82	0.66, 1.02	0.82	0.65, 1.04
Active + Maintenance	39 (0.2)	0.43	0.31, 0.59	0.90	0.59, 1.36	31 (0.2)	0.44	0.30, 0.62	0.88	0.54, 1.43	45 (0.1)	0.45	0.33, 0.61	1.19	0.77, 1.84
**Cardiovascular Events**															
No Treatment	1,186 (0.7)	Referent		Referent		911 (0.7)	Referent		Referent		931 (0.5)	Referent		Referent	
Active Only	46 (0.4)	0.54	0.39, 0.74	0.80	0.57, 1.11	38 (0.4)	0.57	0.40, 0.80	0.93	0.64, 1.36	61 (0.2)	0.53	0.40, 0.71	1.35	0.90, 2.02
Maintenance Only	234 (0.6)	0.84	0.73, 0.97	0.90	0.77, 1.04	180 (0.6)	0.85	0.72, 1.01	0.95	0.80, 1.13	191 (0.4)	0.95	0.80, 1.12	0.95	0.79, 1.13
Active + Maintenance	79 (0.3)	0.50	0.39, 0.64	0.74	0.54, 1.01	54 (0.4)	0.52	0.39, 0.69	0.80	0.55, 1.15	90 (0.2)	0.50	0.40, 0.62	0.94	0.70, 1.26
**HbA1c** ^f^											**Mean ± SD, Beta Estimates**				
No Treatment											7.1 ± 1.3	Referent		Referent	
Active Only											7.5 ± 1.6	0.36	0.20, 0.51	0.04	-0.14, 0.21
Maintenance Only											6.9 ± 1.2	-0.11	-0.22, 0.00	0.01	-0.10, 0.11
Active + Maintenance											7.1 ± 1.4	0.00	-0.13, 0.13	-0.03	-0.16, 0.10

^a^ Coronary artery disease

^b^ Cerebrovascular disease

^c^ Type 2 diabetes

^d^ Number of events that occurred in the follow-up period (percentage of person-months in which an event occurred)

^e^ Adjusted using inverse probability weights (see text and Table 2 for details)

^f^ Glycated hemoglobin

Analyses of HbA1c outcomes in the T2D cohort were restricted to the 3,276 T2D patients with a baseline HbA1c and at least one subsequent measure in follow-up (67.1% of all T2D patients, average of 1.9 follow-up measures per person-year of follow-up). In adjusted models, periodontal therapy was not significantly associated with subsequent HbA1c values. Patients who received active treatment and maintenance care had an average adjusted HbA1c 0.03 percentage points (95% CI: -0.16, 0.10) less than untreated patients (Table 4).

**Fig 1:**
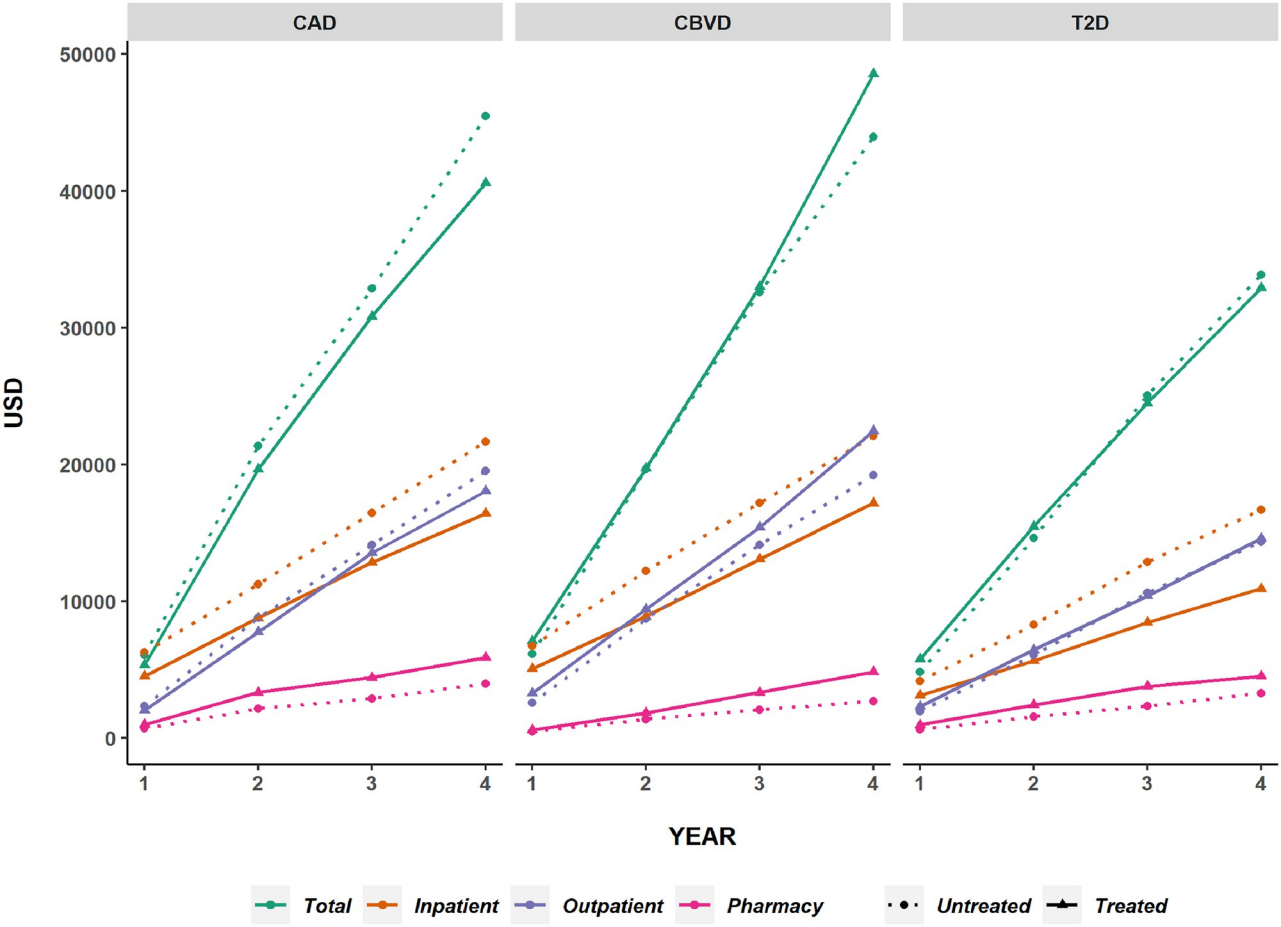
Mean cumulative costs by cohort, year, component and treatment status.

Fig 1 depicts mean cumulative costs by cohort, year, cost component and active treatment status; Table 5 gives adjusted differences in cumulative costs. Cumulative total costs did not differ significantly between groups in any cohort at any time period. Cumulative inpatient costs, however, were significantly lower for treated patients at each year in all cohorts. Conversely, cumulative pharmacy costs were consistently higher in treated versus untreated groups. Over time, 15.9%, 30.5%, and 42.0% of patients were lost to follow up by years 2, 3, and 4, respectively, in the CAD cohort (CBVD: 16.7%, 33.2%, 46.1%; T2D: 18.0%, 33.2%, 44.8%).

**Table 5:** Adjusted differences in mean yearly cumulative costs, treated vs. untreated, by cohort.

	CAD		CBVD		T2D	
	β	95% CI	β	95% CI	β	95% CI
**Year 1**						
**Total Costs**	-707^a^	-1814, 691	956	-689, 3096	931	9, 2029
**Inpatient Costs**	-1737	-2399, -1076	-1667	-2436, -898	-1023	-1379, -667
**Outpatient Costs**	-302	-718, 219	710	-27, 1661	385	25, 811
**Pharmacy Costs**	260	-17, 642	131	-56, 401	322	106, 599
**Year 2**						
**Total Costs**	-1711	-4343, 1327	75	-3063, 3809	826	-877, 2741
**Inpatient Costs**	-2475	-3282, -1669	-3329	-4735, -1924	-2625	-3136, -2113
**Outpatient Costs**	-1036	-2023, 95	695	-660, 2277	378	-276, 1105
**Pharmacy Costs**	1201	214, 2600	476	-128, 1372	857	276, 1622
**Year 3**						
**Total Costs**	-2025	-6119, 2695	407	-5156, 7099	-560	-3305, 2531
**Inpatient Costs**	-3588	-4586, -2591	-4115	-5566, -2665	-4427	-5126, -3728
**Outpatient Costs**	-563	-2222, 1327	1284	-1075, 4070	-243	-1253, 877
**Pharmacy Costs**	1533	53, 3755	1278	52, 3216	1429	404, 2837
**Year 4**						
**Total Costs**	-4917	-10739, 1883	4595	-3925, 14927	-964	-5011, 3652
**Inpatient Costs**	-5252	-6515, -3988	-4932	-6833, -3031	-5768	-6711, -4825
**Outpatient Costs**	-1451	-3825, 1283	3234	-332, 7477	232	-1340, 1995
**Pharmacy Costs**	1928	-211, 5287	2139	181, 5431	1249	-92, 3154

^a^Negative number indicates lower costs in treated versus untreated patients.

## Discussion/Conclusions

We found that patients with periodontitis and CAD, CBVD or T2D who were selected and elected to undergo periodontal treatment and maintenance care in had fewer all-cause hospitalizations, after accounting for confounding by indication for treatment and differential loss to follow-up via inverse probability weighting. Cardiovascular-related hospitalizations, all-cause mortality, cardiovascular events and HbA1c, however, were not consistently associated with selection to treatment status in any cohort. Cumulative total medical care costs did not differ significantly between treated and untreated groups in any disease cohort over 1 to 4 years following baseline. Inpatient care costs were significantly lower over time in treated individuals. These differences were offset, in part, by generally higher outpatient and pharmacy costs in treated individuals.

Overall, published reports of the association between periodontal treatment and clinical medical outcomes are inconsistent. Using a large national (Taiwanese) insurance database, Lin et al. found that periodontal treatment reduced ischemic stroke risk while tooth extractions increased it [36]. Lee et al. analyzed the same national database and reported that risk for myocardial infarction in patients with periodontitis was reduced more following dental prophylaxes (simple cleanings) than “intensive” periodontal treatment, which is counterintuitive given a larger effect was observed with the less intensive treatment [12]. Peng et al. found that among adults with T2D, periodontal treatment was associated with a decreased hazard of myocardial infarction and heart failure, but not stroke [13]. Notably, they found no significant change in the incidence of their composite cardiovascular outcome between treated and untreated groups, which is consistent with our finding. In the current study and compared to untreated individuals, subsequent hospitalizations were lower in those who underwent periodontal treatment with or without follow-up maintenance care, but not in those who received maintenance care alone. These findings suggest that more “intensive” periodontal care is associated with reduced hospitalizations. We found no similar relationship for the other outcomes, however.

We found that periodontal treatment did not significantly lower HbA1c levels in patients with T2D, which is consistent with the largest randomized controlled trial of periodontal treatment in adults with T2D and a non-randomized study of U.S. veterans [37, 38]. Meta-analyses of mostly small RCTs, however, indicate that non-surgical periodontal treatment reduces HbA1c an average of 0.56 percentage points after 6 months [39]. Our findings regarding HbA1c were consistent with the clinical outcomes in this same T2D cohort. Interestingly, Mosen et al. also found that dental treatment was associated with lower rates of all-cause hospitalizations in T2D patients, but not with change in HbA1c [40]. Thakkar-Samtani et al. found that periodontal treatment was associated with reduced overall and outpatient health care costs in T2D patients utilizing Medicaid and commercial insurance claims data [41].

Our findings regarding costs contradict those of Jeffcott et al., who reported significant annual medical care cost reductions in CAD, CBVD and T2D patients associated with periodontal treatment [20]. Whereas Jeffcoat et al.’s study included more individuals than our study, their covariates were limited to age, sex, and T2D status. Their results also have been challenged because only about 1% of their sample were considered treated [42, 43]. Nasseh et al. reported that among individuals with T2D, periodontal treatment was associated with $1577 lower total medical costs and $408 less diabetes-related healthcare costs in years 3 and 4 following diabetes onset [21]. We found slightly lower cumulative total costs with treatment in year 4 ($964, Table 5), although this difference was not significant in our sample. Finally, our results are consistent with two recent European studies. Smits et al. reported nominal medical cost savings (~16 USD in 2021/person/yearly quarter) in Dutch individuals with diabetes following periodontal treatment, while Blaschke et al. found that treatment did not significantly reduce total medical care, inpatient, or diabetes-related drug costs after 3 years in a German cohort [19, 22].

Periodontal disease and edentulism (loss of all teeth) are consistently associated with all-cause and disease-specific mortality [10]. We found that periodontal treatment was not associated with reduced mortality in any cohort, suggesting that periodontitis is not causally linked to mortality. Our sample was not limited to those with severe periodontitis, who are at highest risk for tooth loss. We did, however, consider baseline tooth count and subsequent extractions as covariates in the analyses. We found only two studies that examined the effect of periodontal treatment on mortality [44, 45]. Both were limited to patients undergoing hemodialysis and yielded conflicting results. The larger study analyzed national health insurance claims and found that intensive periodontal treatment significantly reduced mortality, although the periodontal status of individuals was inferred from procedural and not diagnostic codes [44].

### Limitations

Along with previous studies, we did not consider an individual’s response to periodontal treatment. Periodontal treatment improves clinical measures of disease and oral health quality of life, but rarely eliminates all inflammation [46–48]. Poor responders to treatment may be at greater risk for subsequent cardiovascular disease and T2D, [49, 50] and it is possible that periodontal treatment positivity impacts general health outcomes only in patients who respond most favorably to treatment. In the current study, patients were assessed by >100 care providers not specifically trained to measure and record clinical indices in a standardized manner. Thus, we considered only the receipt of treatment as the “exposure.” Finally, there are no widely-accepted and precise criteria to determine treatment success, [51] other than prevention of tooth loss, which tends to occur over decades. We also did not distinguish between treatment provided by a dental specialist (periodontist), general dentist or dental hygienist. Although it is possible that the average response to periodontal treatment may have differed among these provider types, our goal was to examine the effects of periodontal treatment on medical care costs within a healthcare system, and not within provider subtypes. Additionally, non-surgical periodontal treatments and maintenance care are commonly performed in general dental offices, frequently by dental hygienists [52].

We established the 3 cohorts using ICD codes alone, which is a common assessment method in reports of heart disease, stroke, and type 2 diabetes [53–55]. We did not use medication data to identify affected individuals. In a large study of insured US adults with T2D, only 77% were found to be taking diabetes-related medications, suggesting that diagnosis codes, and not medication use, was the more comprehensive assessment method [56]. Nonetheless, an estimated 27–28% of the insured adult population with type 2 diabetes remains undiagnosed. Although our reliance on ICD codes alone may have missed a substantial fraction of those with T2DM, we compared outcomes only among those with an ICD T2D diagnosis, and not between those with and without a T2D diagnosis.

This was not a randomized trial and treated and untreated groups differed substantially in terms of baseline characteristics. Adjustment for covariates markedly attenuated associations seen in the unadjusted analyses of the clinical outcomes. These findings highlight the myriad factors that affect a patient’s decision to undergo treatment [57–59], and the impact of confounders on the relationship between periodontal treatment and non-oral health outcomes. Using simulation, Alshihayb et al. showed that adjusted associations between periodontitis and incident T2D could be further attenuated by considering unmeasured confounders [60]. Other potential confounders include attitudes toward health and oral hygiene, diet and exercise habits, and the dental care provider. We did not have information about the type of dental care provider, including his or her propensity to recommend treatment, and recognize this as a limitation. Information about diet and exercise was available for only a small fraction of our patients and therefore could not be used in the current work. We used IPW to balance treatment groups for confounders, but did not utilize a “doubly robust” approach, which would have produced an unbiased treatment effect estimator if our statistical model (of the causal effect of periodontal treatment on the medical outcomes) was misspecified [61]. Further, our adjusted analyses implicitly assume that that the subgroup of ’no active treatment’ (i.e., ’no care’ and ’maintenance only’) have a similar distribution of confounders for the comparison with ’active treatment’, which may be too strong an assumption. Nonetheless, the substantial differences between unadjusted and IPW (adjusted) results suggest that our models addressed many–but certainly not all–confounders.

Our study included more complete patient data but was considerably smaller than many previous studies. For some outcomes, our samples lacked statistical power to detect small to moderate treatment effects. Although we excluded large treatment effects for some clinical and cost outcomes, larger samples are needed to rule out smaller but potentially clinically meaningful effects.

We examined electronic health record data from 2004–2017. We did not consider the calendar year of an individual’s index date in the analysis. It is possible that, over time, patients with periodontitis are more likely to seek care because of a growing awareness of associations between periodontal disease and other health conditions. Associations between periodontitis and heart disease, stroke, and type 2 diabetes [62–64], however, have been publicized since the early-1990s, which is well before the start of our study period.

Finally, we included only patients with continuous medical and dental insurance one year prior to and at least one year following their index date, but did not have access to measures of socioeconomic status (SES). Insurance plan type, however, was a component of the propensity score for receiving periodontal treatment. Individuals had either comprehensive insurance, which included a benefit for periodontal treatment, or a public plan, which at the time of this study included no such benefit for adults. In this regard, the comprehensiveness of one’s insurance plan might be considered a measure of SES. In 2014–7, only about half of U.S. adults with medical insurance also had dental insurance in the previous 12 months [65]. Individuals without dental insurance are less likely to visit a dentist and undergo treatment [66–68]. Given all individuals in this study had some level of continuous dental insurance, it is unclear why a relatively small fraction of each cohort received any periodontal treatment (see Table 4). Patients accept or refuse periodontal treatment for many reasons, including fear, anxiety, and trust of the provider [69]. Thus, our results may not be fully generalizable to the broader at-risk adult population.

It is not possible to fully elucidate the nature of associations between periodontitis and non-oral diseases through observational studies alone. Patients who pursue periodontal treatment and maintenance care differ from others in a wide variety of characteristics [57, 58, 70], for which statistical adjustments or matching alone are unlikely to fully address. Evidence from large, randomized clinical trials, when feasible, are needed to help determine if periodontal treatment improves medical outcomes and reduces medical care costs in individuals with CAD, CBVD or T2D. Otherwise, studies that use large, comprehensive, and integrated clinical record systems and quasi-experimental observational study designs, such as those that leverage natural experiments or instrumental variables, remain warranted.
